# Endoscopic Assistance in the Deep and Narrow Spaces of the Brain—Microscopic Tumor Surgery Supported by the New Micro-Inspection Tool QEVO® (Technical Note)

**DOI:** 10.3389/fsurg.2021.648853

**Published:** 2021-04-29

**Authors:** Karl-Michael Schebesch, Christian Doenitz, Julius Höhne, Amer Haj, Nils Ole Schmidt

**Affiliations:** Department of Neurosurgery, University Medical Center Regensburg, Regensburg, Germany

**Keywords:** QEVO, KINEVO, micro-inspection tool, brain tumors, ventricle tumors, parasellar area, cerebello-pontine angle, endoscopic neurosurgery

## Abstract

**Introduction:** To evaluate the feasibility and efficacy of the innovative micro-inspection tool QEVO® (Carl Zeiss Meditec, Oberkochen, Germany) as an endoscopic adjunct to microscopes for better visualization of the surgical field in complex deep-seated intracranial tumors in infants and adults.

**Materials and Methods:** We retrospectively assessed the surgical videos of 25 consecutive patients with 26 complex intracranial lesions (time frame 2018–2020). Lesions were classified according to their anatomical area: 1 = sellar region (*n* = 6), 2 = intra-ventricular (except IV.ventricle, *n* = 9), 3 = IV.ventricle and rhomboid fossa (*n* = 4), and 4 = cerebellopontine angle (CPA) and foramen magnum (*n* = 7). Indications to use the QEVO® tool were divided into five “QEVO® categories”: A = target localization, B = tailoring of the approach, C = looking beyond the lesion, D = resection control, and E = inspection of remote areas.

**Results:** Overall, the most frequent indications for using the QEVO® tool were categories D (*n* = 19), C (*n* = 17), and E (*n* = 16). QEVO® categories B (*n* = 8) and A (*n* = 5) were mainly applied to intra-ventricular procedures (anatomical area 2).

**Discussion:** The new micro-inspection tool QEVO® is a powerful endoscopic device to support the comprehensive visualization of complex intracranial lesions and thus instantly increases intraoperative morphological understanding. However, its use is restricted to the specific properties of the respective anatomical area.

## Introduction

In modern neurosurgery, the rationale behind pre- and intraoperative decision-making is complete removal of the intracranial tumor *and* preservation of neurological function. Lesions inside the cerebellopontine angle (CPA) or close to or in the foramen magnum as well as tumors in the rhomboid fossa or within the ventricles have a potential strong anatomical relationship with various vulnerable cranial nerves, significant vasculature, and eloquent areas. Particularly in the case of complex tumors at the skull base, such lesions require not only extensive surgical skills and clinical experience but also an extensive technical armamentarium. For this reason, such patients—many of them children or infants—are predominantly treated at high-volume tertiary neuro-oncology centers.

Over the past decades, technical innovations in direct and indirect intraoperative visualization have significantly changed surgical approaches as well as dissection strategies. Among these visual adjuncts, neuro-endoscopes have become well-established tools, particularly in the transsphenoidal surgery of intra- and parasellar lesions (1–3) at the anterior skull base (4–9) or for alternative drainage in occlusive hydrocephalus (2, 10, 11).

However, the intraoperative use of a surgical endoscope is elaborate because it is necessary to drape the endoscope and to supply the operating theater with additional machines and monitors, which is a time-consuming and expensive process. Most neurosurgical endoscopes have a fixed angle of 0° or 30°, rendering the “look around the corners” complicated for the surgeon. Furthermore, the employment of conventional surgical endoscopes add another dimension of complexity in handling when used to assist microscopic-guided surgery.

In microscope-guided surgeries of complex deep-seated tumors in the narrow spaces of the brain, surgeons require all possibly available information about the environment of the targeted lesion. Such information enables surgeons to tailor the approach, to visualize hidden vessels and nerves, to inspect remote areas, and to control the resection status in more detail. A small handheld endoscope—that can be easily connected to the monitor of the microscope without any additional hardware and without the necessity of having to drape it—would deliver such crucial information within seconds without substantially impeding the surgical workflow.

Here, we present our experiences in applying the innovative micro-inspection tool QEVO® (Carl Zeiss Meditec, Oberkochen, Germany) in complex neuro-oncologic procedures. We discuss the indications, benefits, and limitations of this small plug-and-play endoscope and describe a few illustrative cases classified according to the anatomical area of the lesion in the brain.

## Materials, Patients, and Methods

**Figure 1:**
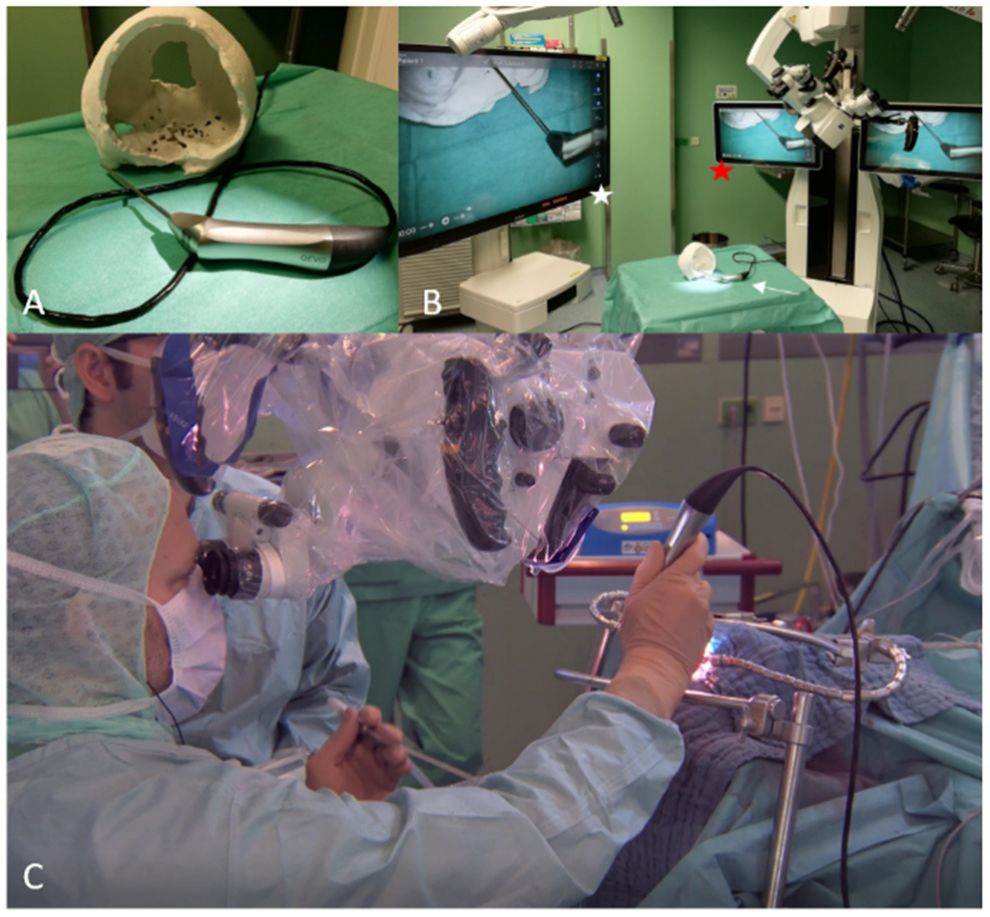
QEVO®, KINEVO®, and monitors in the Operating Room. **(A)** QEVO® micro-inspection tool. **(B)** QEVO® (white arrow) connected to KINEVO® surgical microscope (red asterisk) and to the external monitor (white asterisk). **(C)** Simultaneous use of QEVO® and KINEVO®.

The micro-inspection tool QEVO® has been described previously (12). This handheld endoscope measures 12 cm in length and 3.6 mm in thickness and weights 250 g. The angle of view is fixed at 45°, and illumination is provided by an integrated light source. The QEVO® tool is re-sterilizable, does not need to be draped, and is directly connected to the KINEVO® surgical microscope (Carl Zeiss Meditec, Oberkochen, Germany). The real-time 4K image is displayed either on the internal monitor of the microscope or on an external monitor, or both. The images can also be directly displayed through the oculars of the KINEVO® (see Figure 1).

All operations were conducted microsurgically with the KINEVO® surgical microscope, equipped with the QEVO® micro-inspection tool. All patients except for patients #12 and #24 underwent standardized open craniotomy. In patients #12 and #24, the tumors were located in the proximal cervical spine and thus approached via laminotomy of C1. Each full procedure was stored on the internal and an external hard drive. For the purpose of this study, all videos were reviewed by the author and his team.

Consistently, all operations had been microscopically performed, and if required, the QEVO® tool was briefly and repeatedly inserted into the surgical field for the following purposes (QEVO® categories): (A) to exactly locate the lesion if not directly depicted with the microscope; (B) to tailor the approach; (C) to look beyond structures to visualize adjacent tissues, vessels, and nerves, or to define the resection borders; (D) to control the grade of resection; and (E) to inspect remote areas and spaces (e.g., bleeding control in the ventricles and subdural spaces). Surgical maneuvers were exclusively conducted under the microscope, and the QEVO® tool was solely used to provide essential information.

The tumors were allocated to groups according to the four main areas of the brain: (1) supra- and parasellar and pre-chiasmatic (*n* = 6), (2) intra-ventricular (lateral ventricles and III.ventricle) (*n* = 7), (3) IV.ventricle and rhomboid fossa (*n* = 4), and (4) CPA, foramen magnum, and cranio-cervical junction (*n* = 7). The huge infratentorial epidermoid (patient #3) was allocated to groups 3 and 4 because of its size. A case overview with baseline data is presented in Table 1.

**Table 1:** Overview and baseline data of patients.

**Pat**	**Age**	**Sex**	**Confirmed histopathology**	**Anatomical area**	**Grade of resection**	**New neurological deficits**	**QEVO^®^ category**
1	3	m	Craniopharyngeoma	1	GTR	No	C,D
2	2	m	Ependymoma WHO III	3	GTR	No	B,C,D
3	41	m	Epidermoid	3,4	GTR	No	A,C,D,E
4	51	m	Gangliocytoma WHO I	2	GTR	No	D,E
5	1	f	Pilocytic astrocytoma WHO I	2	STR	No	B,C,D,E
6	38	f	Meningioma WHO I	1	GTR	No	C,D
7	43	m	Colloid cyst	2	GTR	No	B,C,D,E
8	55	m	Subependymoma WHO I	2	GTR	No	B,C,D,E
9	60	f	Dermoid	3	GTR	No	E
10	15	m	Craniopharyngeoma	2	STR	No	C,D
11	36	m	Giant cell astrocytoma WHO I	2	GTR	No	A,B
12	46	f	Meningioma WHO I	4	GTR	No	A,C,E
13	55	m	Acoustic neuroma	4	GTR	Postoperative hemorrhage, moderate facial palsy	C,D,E
14	33	m	Chordoid glioma WHO II	2	STR	Cognitive decline	B,C,D,E
15	54	m	Meningioma WHO I	1	GTR	No	C,D
16	43	f	Hemangiopericytoma WHO II	4	GTR	Hoarseness	C,E
17	48	f	Pilocytic astrocytoma WHO I	2	GTR	No	A,B,D,E
18	58	m	Pituitary adenoma	1	STR	Diabetes insipidus	C,D
19	11	m	Craniopharyngeoma	1	STR	No	C,D
20	71	f	Meningioma WHO I	1	GTR	No	D,E
21	70	f	Meningioma WHO I	4	GTR	No	D,E
22	53	f	Meningioma WHO I	4	GTR	No	A,B,C,D,E
23	14	m	Teratoma	2	GTR	No	D
24	34	f	Ependymoma WHO III	4	GTR	No	C,E
25	6	f	Pilocytic astrocytoma WHO I	3	GTR	No	E

*Pat means patient, f means female, m means male, WHO means world health organization; CSF means cerebrospinal fluid; GTR means gross total resection; STR means subtotal resection*.

*QEVO® category: A, target localization; B, tailoring the approach; C, looking beyond; D, resection control; E, inspection of remote areas*.

*Anatomical area: 1, parasellar and pre-chiasmatic; 2, ventricular (lateral ventricle, III.ventricle); 3, rhomboid fossa, IV ventricle; 4, foramen magnum, cranio-cervical junction*.

Each patient was retrospectively evaluated regarding the time point at which the QEVO® tool had been applied and what additional visual information had been generated. Based on this information, the patient was allocated to one of the five QEVO® categories (A–E).

The study was approved by the internal review board (Ethics Committee of the University of Regensburg, AZ 20-1951-104, 22 July 2020).

## Results

Twenty-five patients with complex deep-seated intracranial lesions or lesions within the proximal cervical spine were included in this series (time frame 2018–2020). Mean age was 37.6 years (range 1–71 years), 11 patients were women, and 7 patients were children (1 to 15 years).

The micro-inspection tool QEVO® was used in each operation but never longer than 10 min in total per patient. The endoscopic information additionally provided by the QEVO® tool varied substantially between the patients. In 19 patients, the tool was used for final resection control (category D). In 17 patients, the tool was used to look beyond the tumor to detect any adhesive nerves, vessels, and tissue (category C) during resection. In 16 patients, the ventricles and subdural spaces were endoscopically inspected to rule out any relevant bleeding within these remote areas (category E). In eight patients, the approach was modified and tailored according to the endoscopic findings (category B), and in five patients, the tool was used to detect the tiny and hidden targeted lesion (category A). Further details are presented in Table 1 and Figure 6.

Insertion of the micro-inspection tool QEVO® was always feasible without any technical issues, and its image quality was excellent at all times. We did not encounter any QEVO®-related complication or morbidity in any of our 25 patients. Complete resection (GTR) was achieved in all patients but patients 5, 10, 14, 18, and 19.

To outline the most important aspects of the visual benefits of the micro-inspection tool QEVO®, we chose representative cases for each of the four predefined anatomical areas (1–4) and for each of the five QEVO® categories (A–E).

### Illustrative Cases

**Figure 2:**
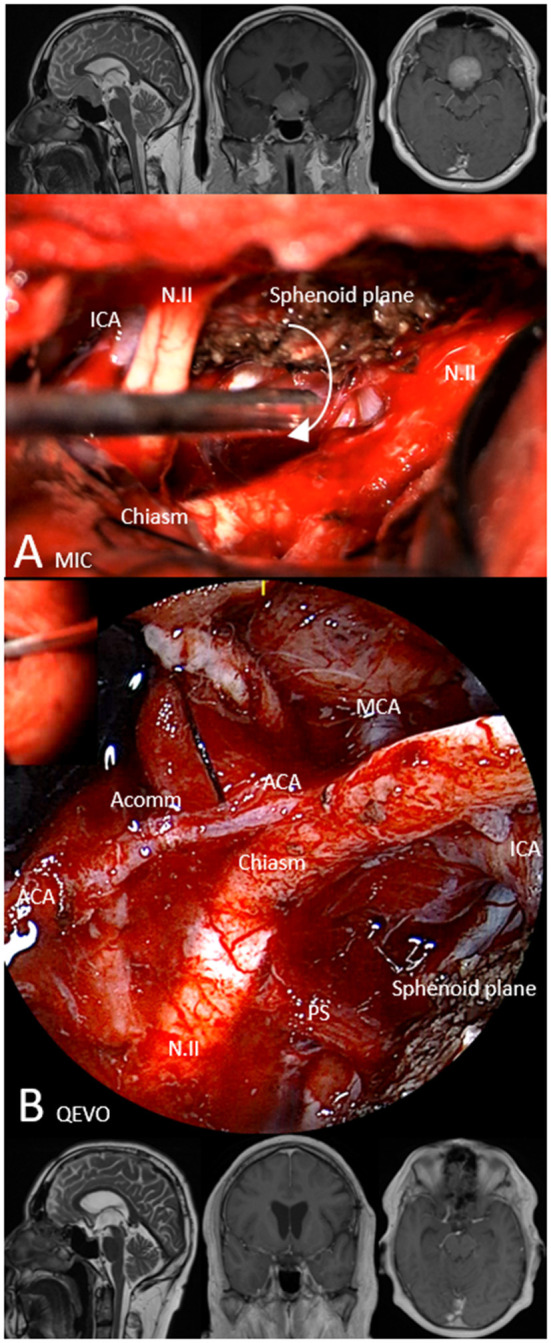
Illustrative case 1—area 1, QEVO® category D. Patient #15, meningioma WHO I of the sphenoid plane, left pterional approach. Upper row, Preoperative MRI in sagittal plane (T2), coronal plane, and axial plane (T1 with contrast medium); **(A)** MIC(roscopic), Microscopic image at the end of tumor resection. The white arrow indicates the position and visual trajectory for the micro-inspection tool QEVO® **(B)**; **(B)** QEVO, Endoscopic image enabling the view below the tumor-free optic chiasm and the pituitary stalk, depicting the arteries of the anterior circulation. Lower row, Postoperative MRI in sagittal plane (T2), coronal plane, and axial plane (T1 with contrast medium). The white arrow indicates the visual trajectory for micro-inspection tool QEVO®, ICA means Internal Carotid Artery, N.II, Optic nerve, MCA means Middle Cerebral Artery, ACA means Anterior Cerebral Artery, Acomm means Anterior Communicating Artery, PS means Pituitary Stalk.

Figure 2. Brief case description and surgical strategy:

A 54-year-old man presented with visual disturbances in the left eye. Magnetic resonance imaging (MRI) showed an intra- and suprasellar mass, highly suspicious of meningioma of the sphenoid plane that affected the optic nerves and chiasm. The meningioma was dissected and removed in a piecemeal fashion using a standardized left-sided pterional approach. The dural adhesion at the median sphenoid plane was resected and covered with a piece of collagen. Because microscopic evaluation (Figure 2A) could not exclude any tumor remnants below the chiasm or optic nerves, the micro-inspection tool QEVO® was used to depict the tumor-free region below the chiasm (Figure 2B). Hereby, the preserved pituitary stalk and large parts of the anterior circulation came into view. Surgery was completed, and the patient was transferred to the neurosurgical intensive care unit (ICU) where he woke up immediately. The postoperative clinical course was uneventful, and postoperative MRI showed no tumor residuals. After 12 months, the visual disturbances had completely resolved.

**Figure 3:**
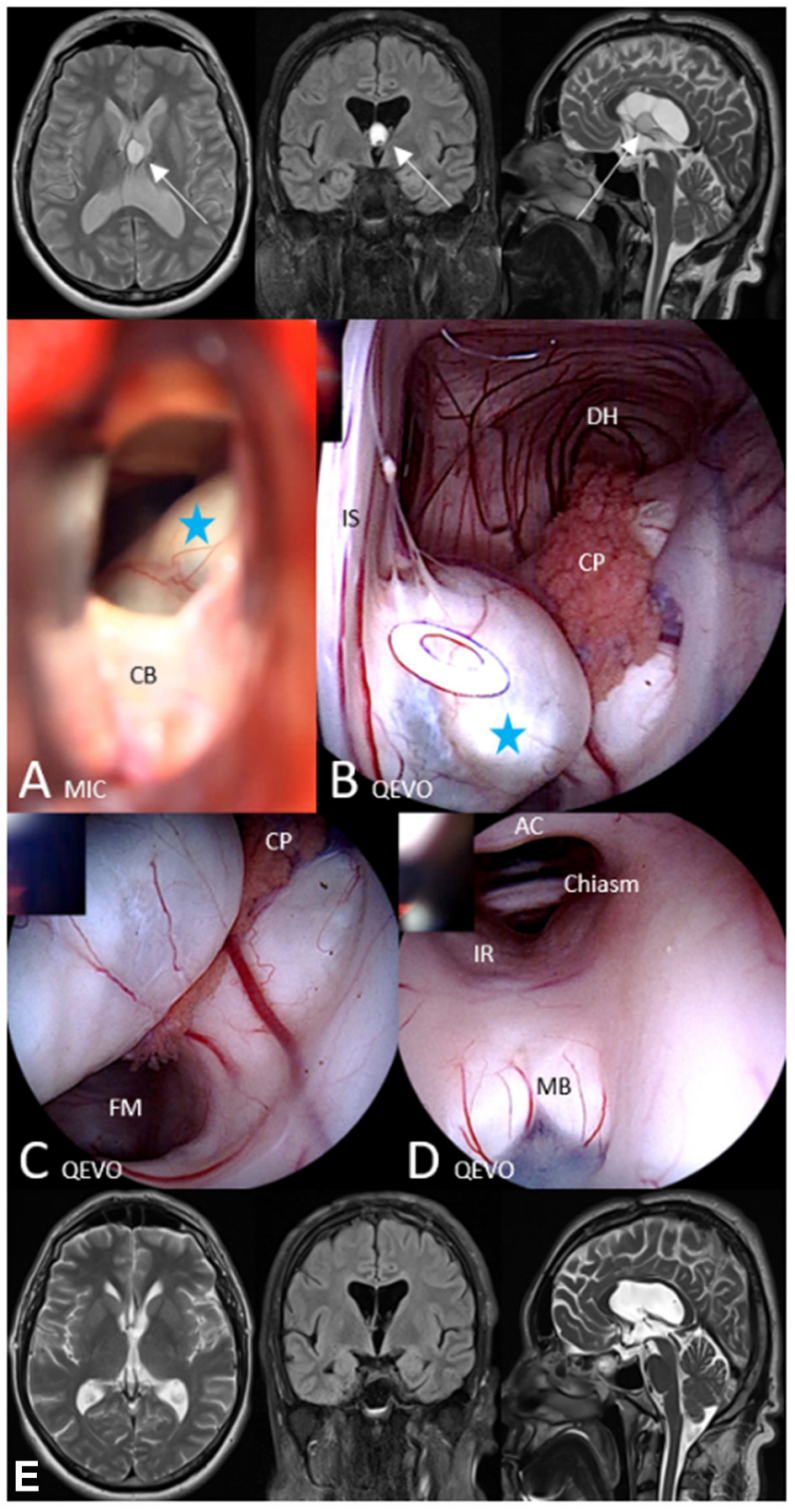
Illustrative case 2—area 2, QEVO® categories **(A–E)**. Patient #7, colloid cyst at the foramen of Munro, left-sided transcallosal approach. Upper row, Preoperative MRI in axial plane (Flair), coronal plane (T1), and sagittal plane (T2); A MIC(roscopic), After opening of the callosal body, view at the partially hidden cyst. B QEVO, Inspection of the dorsal horn and related structures; C QEVO, View below the colloid cyst with open foramen of Munro of the right lateral ventricle; D QEVO, Inspection of the third ventricle with related structures. **(E)** Lower row, Postoperative MRI in axial plane (T2), coronal plane (T1), and sagittal plane (T2). White arrows indicate the colloid cyst on the preoperative MRI, blue asterisk, colloid cyst, CB means Callosal Body, DH means Dorsal Horn, CP means Choroid Plexus, FM means Foramen of Munro, IR means Infundibular Recessus, MB means Mamillary Bodies, AC means Anterior Commissure.

Figure 3. Brief case description and surgical strategy:

A 43-year-old man had developed severe refractory headache. MRI showed a colloid cyst at the foramen of Munro with consecutive moderate hydrocephalus. During left paramedian craniotomy, the frontal callosal body was exposed using the interhemispheric approach, and a small hole was created in the midline (Figure 3A). The cyst was partially visible, and the micro-inspection tool QEVO® was inserted to exactly visualize the proportion of the cyst (Figure 3B). Callosotomy was tailored accordingly. Before resection of the cyst was started, we checked by means of further endoscopic evaluation if any adherences were present within the foramen of Munro or within parts of the choroid plexus (Figure 3C). After en bloc removal of the cyst, the QEVO® tool was passed through the foramen of Munro to inspect the III.ventricle to rule out any accumulation of blood in the frontal or dorsal parts (Figure 3D). Surgery was completed, and the patient woke up immediately. MRI conducted on the next day showed neither any residuals of the cyst nor any hemorrhage within the ventricles. The further clinical course was uneventful, and the headache had completely resolved within a few days.

**Figure 4:**
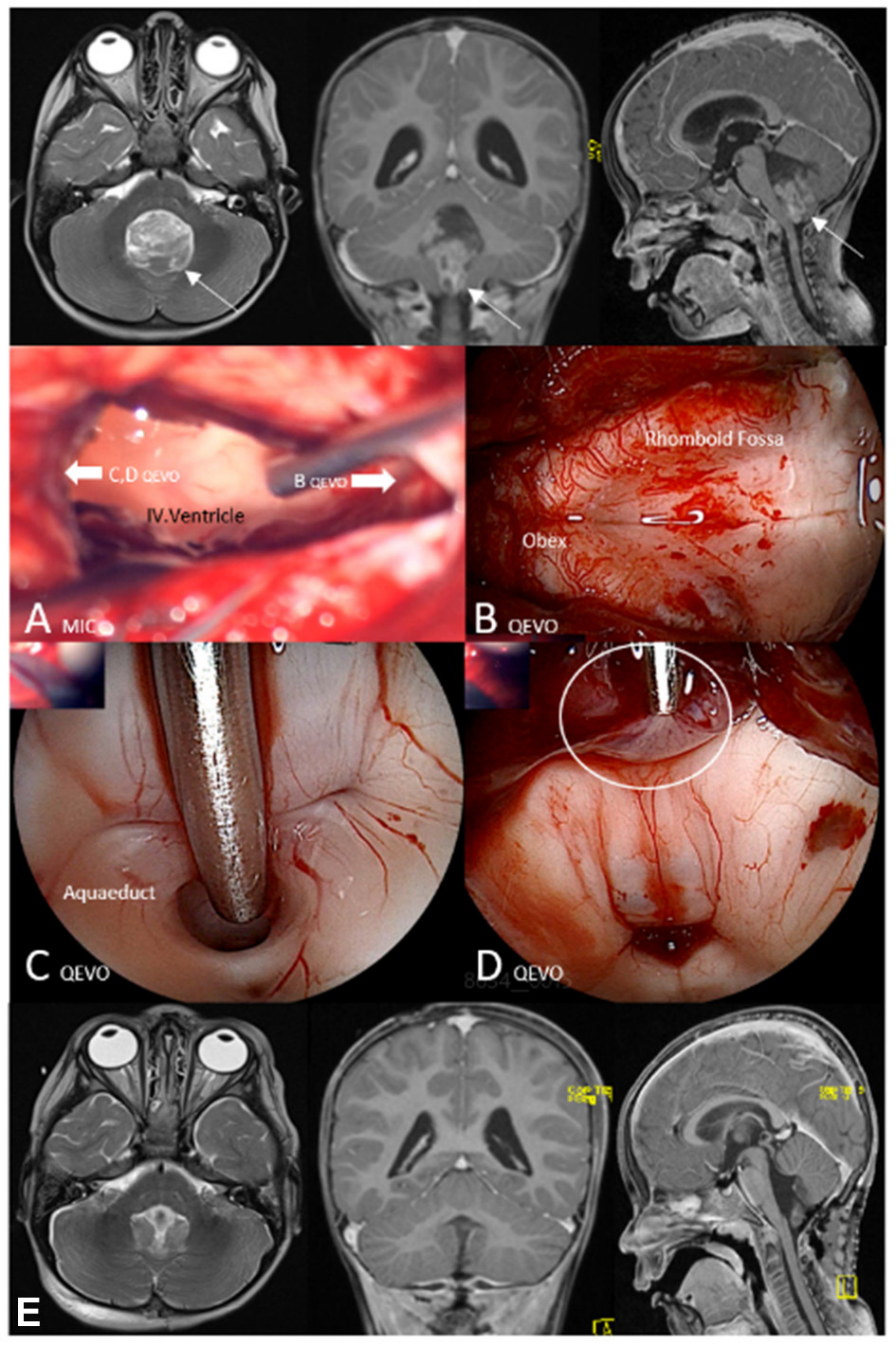
Illustrative case 3—area 3, QEVO® categories **(D, E)**. Patient #2, anaplastic ependymoma WHO III, median suboccipital approach. Upper row, Preoperative magnetic resonance image (MRI) in axial plane (T2), coronal plane, and sagittal plane (T1 with contrast medium); **(A)** MIC(roscopic), View at the IV.ventricle after tumor resection; the white arrows indicate the visual trajectories of the micro-inspection tool QEVO® **(B–D)**; **(B)** QEVO, Inspection of the dorsal horn and related structures; **(C)** QEVO, Endoscopic view at the aquaeductus cerebri; **(D)** QEVO, Tumor remnant at the roof of the ventricle (white circle); **(E)** Lower row, Postoperative MRI in axial plane (T2), coronal plane, and sagittal plane (T1 with contrast medium). White arrows indicate the tumor within the IV ventricle on the preoperative MRI; the white circle indicates a tumor remnant at the roof of the ventricle.

Figure 4. Brief case description and surgical strategy:

A 3-year-old boy presented at the pediatric department with nausea and vomiting. MRI showed massive hydrocephalus due to a huge inhomogeneous mass within the IV.ventricle, strongly suspicious for ependymoma. Median suboccipital craniotomy was conducted with the child in prone position. The tumor was identified immediately after durotomy. After removal of the ventricular portion of the ependymoma, the IV.ventricle was inspected microscopically (Figure 4A) and endoscopically with the QEVO® tool that was directed toward the obex (Figure 4B) to visualize the rhomboid fossa and toward the aqueduct (Figure 4C) to visualize the ventricle's roof. Identification and, consequently, removal of the apical tumor remnants were only possible by using the micro-inspection tool QEVO® (Figure 4D). Surgery was completed, and the boy was transferred to the pediatric ICU. After an early MRI scan, the patient was extubated the next day. The postoperative MRI scan was evaluated to be tumor-free, and adjuvant treatment was planned accordingly. The further clinical course was uneventful, no new deficits occurred, and wound healing was unaffected.

**Figure 5:**
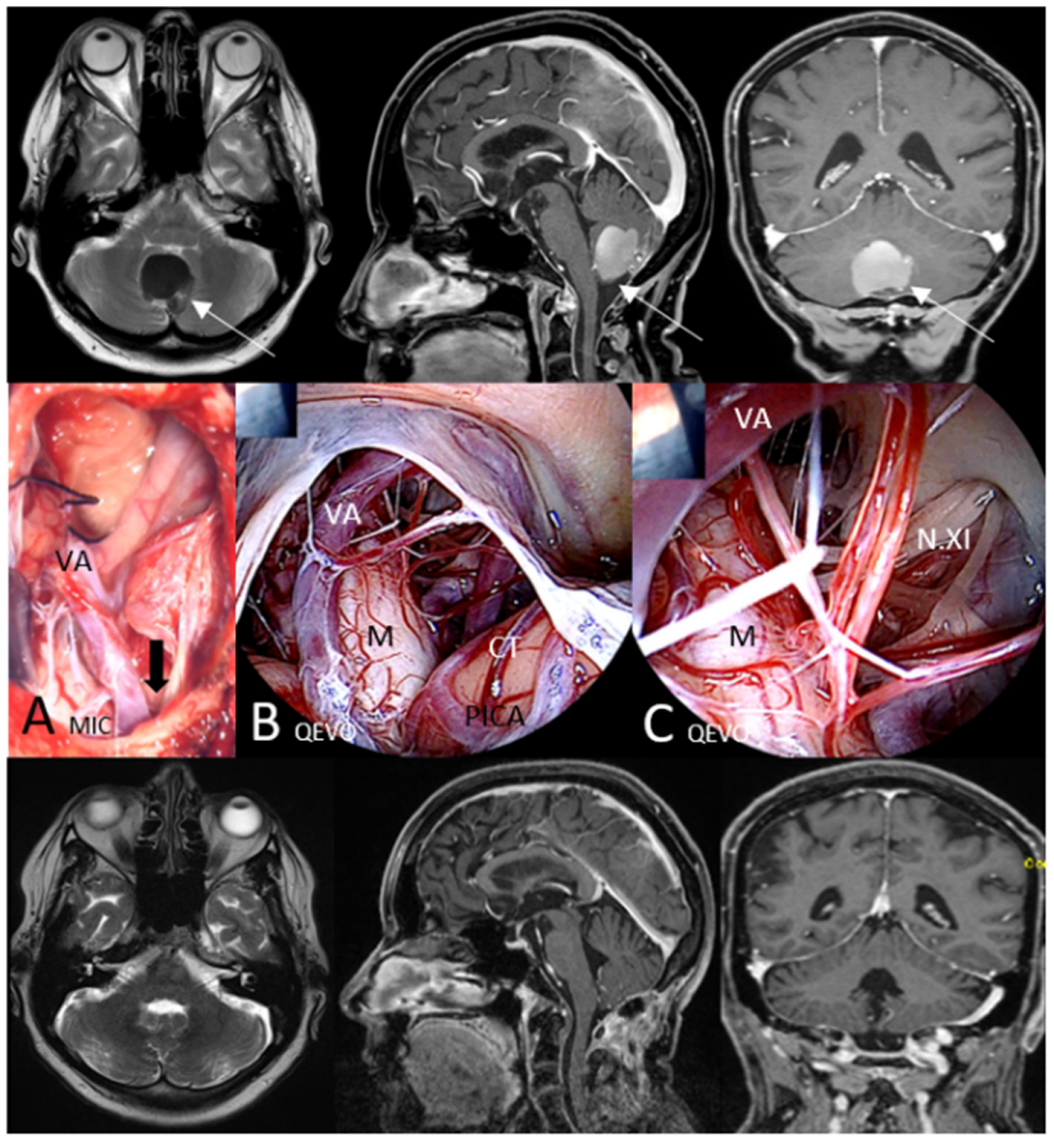
Illustrative case 4—area 4, QEVO® category E. Patient #9, cerebellar dermoid at the level of the foramen magnum, median suboccipital approach. Upper row, Preoperative MRI in axial plane (T2), sagittal plane, and coronal plane (T1 with contrast medium); **(A)** MIC(roscopic), View at the resection cavity at the level of the open foramen magnum; the black arrow indicates the visual trajectory of the QEVO® **(B, C)**; **(B)** QEVO, Inspection of the cranio-cervical junction with related structures; C QEVO, Deeper and closer view on the medulla oblongata with lower cranial nerves exiting the spinal canal; Lower row, Postoperative MRI (3 months after surgery) in axial plane (T2), sagittal plane, and coronal plane (T1 with contrast medium). White arrows indicate the tumor in the preoperative MRI, VA means Vertebral Artery, M means Medulla oblongata, CT means Cerebellar Tonsilles, PICA means Posterior Inferior Cerebellar Artery, N.XI, spinal Accessory Nerves.

Figure 5. Brief case description and surgical strategy:

A 60-year-old woman had developed headache, nausea, and vomiting. MRI showed a huge infratentorial mass within the lower cerebellum close to the foramen magnum. The patient underwent median suboccipital craniotomy and opening of the foramen magnum in prone position. The lesion was analyzed as dermoid cyst and completely removed microscopically (Figure 5A). To rule out any relevant bleeding or undetected satellites, the QEVO® tool was used to provide an overview of the medullary parts below the foramen magnum (Figure 5B) and to inspect the lateral parts of the cranio-cervical junction in more detail (Figure 5C). Surgery was completed, and the patient woke up immediately without displaying any neurological deficits. Wound healing was uneventful, and MRI 3 months after surgery showed the surgical cavity to be tumor-free.

## Discussion

Historically, the most important indications for endoscopic cranial neurosurgery are transsphenoidal or subfrontal approaches to the sellar region and to the anterior skull base (2, 13). A further indication in patients with occlusive hydrocephalus is the approach to and the opening of the floor of the third ventricle (10, 11). Over the past few years, surgical endoscopes have undergone consistent technical refinement, especially with regard to easier handling and improved image transmission in terms of full-HD and three-dimensional visualization. These technical innovations have largely increased the number of scientific reports on the use of endoscopes in vascular and oncologic neurosurgery of the brain and spine (4, 8, 14–18).

One of the most innovative handheld surgical endoscopes is the micro-inspection tool QEVO® that has been recently evaluated as a highly useful device in aneurysm microsurgery (18). This tool, measuring 12 cm in length and weighing 250 g, is equipped with an internal light source and has a fixed angle of 45°. Technical details are described elsewhere (12).

This handheld surgical micro-inspection tool QEVO® cannot replace a surgical microscope, but represents a further innovation in endoscopic-assisted microsurgery as its intuitive use potentially amplifies and completes visual impression for the neurosurgeon, resulting in a more comprehensive anatomical understanding of the surgical field and its environment. After a short learning curve, this tool is easy to use as an undraped plug-and-play device that can be easily connected to the KINEVO® visualization platform. For these reasons, the micro-inspection tool QEVO® has become a regular feature at our department since its invention in 2018.

The cumulative use duration of the QEVO® tool rarely exceeds 5–10 min per operation. However, this tool provides valuable details for successful surgery, for instance information on the target site, hidden or concealed adhesions with vulnerable tissues, nerves and vessels, the grade of resection, hemorrhages, or the accumulation of blood in remote areas such as the ventricle horns or the subdural spaces. These advantages help to achieve low procedure-related morbidity in complex brain surgery. The presented clinical cases illustrate the visual benefits provided by this small endoscope. However, some relevant technical limitations of the QEVO® tool should be addressed in the future, for instance, the integration of fluorescent filters, a modifiable angle of view, and the possibility to fix the tool to the head clamp so that surgeons have both hands free.

Obviously, not every additional endoscopic information was feasible or even useful in the different types of surgeries. Consequently, our motivation for this study was to present an overview about the variable rationale for the use of the QEVO® tool, prioritized for different paradigms, i.e., the “QEVO® categories”: (A) Is the tool helpful in finding tiny hidden tumors? (B) Does its use result in modifying the surgical approach toward the lesion? (C) Is it possible to gather relevant information on the area behind or below the tumor? (D) Can it improve the surgeon's appraisal of the grade of resection in the final check? (E) Can remote areas be easily inspected to rule out any relevant bleeding that may have been overlooked microscopically? Evaluation of the surgical videos of the 25 patients showed that resection control (D), looking beyond the tumor (C), and inspection of the remote areas (E) were the most common indications for using the QEVO® tool. The tool was less frequently applied for detecting lesions (A) and for tailoring the approach (B). However, in intra-ventricular lesions that are difficult to access, the angled view of the tool has proven to be extremely helpful because it allows to evaluate the site of the lesion through a tiny hole and to accordingly modify the access through the callosal body (see patient #2).

Because of the retrospective character of this series including adults and infants with complex deep-seated lesions, it did not seem reasonable to assess the frequency of a changed surgical strategy according to the QEVO® image. Although this omission is a limitation of this study, it could not be reliably determined retrospectively how and when the surgical strategy was adopted with relation to endoscopic visualization.

**Figure 6:**
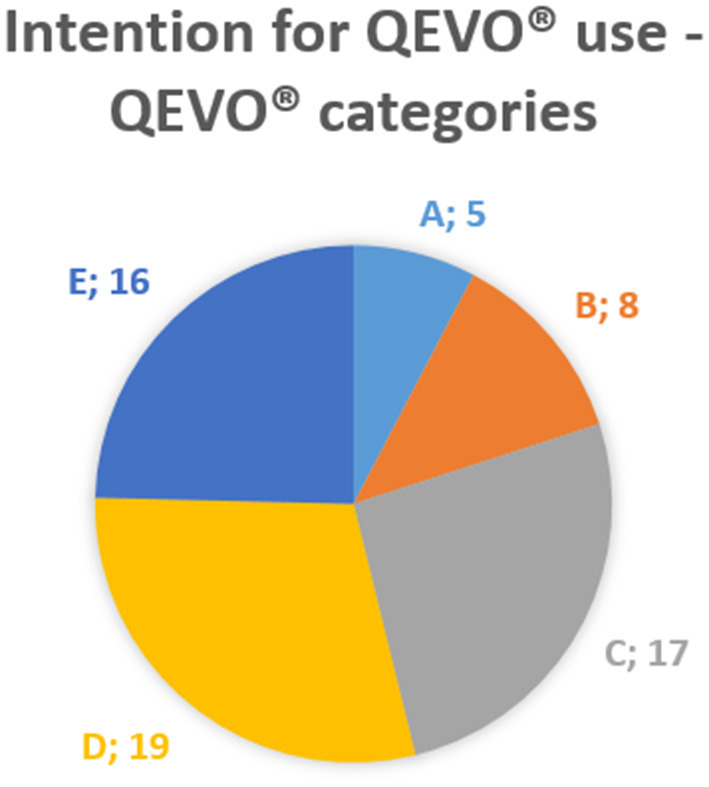
QEVO® categories. A, target localization; B, tailoring the approach; C, looking beyond; D, resection control; E, inspection of remote areas.

The subdivision of the 25 patients according to the four anatomical areas affected reflects the effort of the authors to distinguish different grades of surgical complexity with regard to the surgical approach and dissection, which implicates a different use of the endoscope. Not surprisingly, Table 1 and Figure 6 show the broad variation in the application of the QEVO® tool in relation to the specific properties of the anatomical areas. For parasellar tumors (anatomical area 1), the micro-inspection tool was never used for detecting the tumor (A) or for tailoring the approach (B). In contrast, the QEVO® tool was frequently used for tailoring the approach (B) in intra-ventricular tumors (anatomical area 2). Irrespective of the anatomical area, five tumors were not removed completely. In these patients, resection control with the micro-inspection tool confirmed the tumor remnants, but surgery was stopped intentionally.

In summary, the application of this endoscopic tool was relevant for generating additional anatomical information about the tumor and its environment, at least in this patient series. Many authors, who evaluated the potential benefit of endoscopic-assisted surgery, have outlined the value of the additional endoscopic field of view (4, 7, 9, 12–14). Especially in deeply located areas of the brain, such a technical adjunct can strongly increase the safety and mastery of the surgical procedure, even when its cumulative duration of use does not exceed a few minutes. The micro-inspection tool QEVO® is very easy to use, offers intuitive handling, does not prolong the duration of surgery, and does not require the use of additional monitors or other armamentarium. However, some technical limitations should be addressed to further increase the tool's value for surgery.

## Conclusion

In the conventional microscopic neurosurgery of complex deep-seated cranial tumors, the additional use of the handheld, 45°-angled, plug-and-play micro-inspection tool QEVO® provides relevant information for the safe approach, dissection, and removal of lesions. The frequency and indications for using this endoscopic tool depend on the specific properties of the targeted anatomical area.

## Data Availability Statement

The data analyzed in this study is subject to the following licenses/restrictions: Surgical videos stored on external hard-drive. Requests to access these datasets should be directed to karl-michael.schebesch@ukr.de.

## Ethics Statement

The studies involving human participants were reviewed and approved by Ethics Committee of the University of Regensburg, Germany. Written informed consent from the participants' legal guardian/next of kin was not required to participate in this study in accordance with the national legislation and the institutional requirements.

## Author Contributions

KMS designed and wrote the manuscript and surgeon of nearly all presented cases. CD, JH, AH, and NS critically revised the manuscript, added images, and gave scientific input. All authors contributed to the article and approved the submitted version.

## Conflict of Interest

KMS and JH have received honoraria, travel fees and financial support from Carl Zeiss Meditec, Germany for previous studies. The remaining authors declare that the research was conducted in the absence of any commercial or financial relationships that could be construed as a potential conflict of interest.
